# Characterization of the Effects of Mesenchymal Stromal Cells on Mouse and Human Islet Function

**DOI:** 10.1002/sctm.19-0023

**Published:** 2019-05-08

**Authors:** Ahmed A. Arzouni, Andreia Vargas‐Seymour, Paramjeet K. Dhadda, Chloe L. Rackham, Guo‐Cai Huang, Pratik Choudhary, Aileen J. F. King, Peter M. Jones

**Affiliations:** ^1^ Department of Diabetes School of Life Course Sciences, King's College London London United Kingdom

**Keywords:** Mesenchymal stromal cell, Islet, β‐Cell, Insulin secretion

## Abstract

Islet transplantation has the potential to cure type 1 diabetes, but current transplantation protocols are not optimal and there is extensive loss of islet β‐cell insulin secretory function during the immediate post‐transplantation period. Studies using experimental models of diabetes have shown that the coculture of islets with mesenchymal stromal cells (MSCs) prior to transplantation improves graft function, but several variables differed among research groups (e.g., type of MSCs used and the treatment conditions). We have therefore assessed the effects of MSCs on mouse and human islets by investigating the importance of tissue source for MSCs, the coculture protocol configuration and length, the effect of activated MSCs, and different β‐cell secretory stimuli. MSCs derived from adipose tissue (aMSCs) were the most effective at supporting β‐cell insulin secretion in both mouse and human islets, in a direct contact coculture configuration. Preculture with aMSCs enhanced both phases of glucose‐induced insulin secretion and further enhanced secretory responses to the non‐nutrients carbachol and arginine. These effects required a coculture period of 48–72 hours and were not dependent on activation of the MSCs. Thus, direct contact coculture with autologous, adipose‐derived MSCs for a minimum of 48 hours before implantation is likely to be an effective addition to human islet transplantation protocols. stem cells translational medicine *2019;8:935&944*

**Significance Statement**

It is well established that mesenchymal stromal cells (MSCs) have beneficial effects on the functional survival of islet grafts in experimental models of type 1 diabetes, and studies from different groups have demonstrated that pretreating isolated islets with MSCs is sufficient to improve graft function. However, these studies differ in the sources of MSCs, MSC/islet coculture protocol configuration and length, and other variables. The authors have therefore assessed the importance of some of these variables in coculture of mouse and human islets with MSCs to define how best to modify human islet transplantation protocols.

## Introduction

Islet transplantation is gaining acceptance as a therapy for type 1 diabetes (T1D), but current protocols are not optimal 1: there is extensive loss of islet β‐cell insulin secretory function during the pretransplantation culture in vitro 1, 2 and during the immediate post‐transplantation period, when islet function and survival are compromised by the hypoxic, inflammatory host environment 3. Mesenchymal stromal cells (MSCs) offer the potential for improving the outcomes of islet transplantation by exerting anti‐inflammatory, immunosuppressant, and regenerative effects to protect the islet graft 4, 5, 6, 7. Several studies have demonstrated that cotransplantation of MSCs with islets has beneficial effects on graft function to maintain normoglycemia in animal models of T1D 8, 9, 10, 11, 12. Some of these effects can be attributed to MSCs influencing the host niche to reduce inflammation 13, 14, 15, to suppress acquired immune responses 4, and to enhance graft revascularization 8, 16. Moreover, in vitro studies have demonstrated that MSCs also exert direct effects on β‐cells to enhance glucose‐induced insulin secretion 17, 18, 19 and to protect them from inflammatory cytokine‐induced apoptosis 20. These effects can be attributed, at least in part, to MSC‐derived soluble mediators 20 and extracellular matrix (ECM) 17 and may be dependent on, or modified by, the activation of MSCs by inflammatory cytokines 4, 5, 21. Our previous studies in a mouse model of islet transplantation have demonstrated that these effects on islet function in vitro translate into improved glycemic control by the islet graft in vivo 18, 19, 20.

These observations raise the possibility of improving transplantation outcomes by pretreating the islet graft material before implantation to ensure robust insulin secretion post‐transplantation. However, little attention has been paid to define how best to pretreat islets with MSCs to maintain their optimal function. In this study, we have assessed the functional effects of coculturing mouse and human islets with MSCs and have investigated the importance of MSC tissue source, MSC/islet coculture configuration and length, MSC activation, and the β‐cell secretory stimulus.

## Methods

### Islet Isolation and Culture

Mouse islets were isolated by collagenase digestion of mouse pancreas followed by density gradient separation (as previously described 22), and maintained in culture (37°C, 5% CO_2_) in Roswell Park Memorial Institute (RPMI)‐1640 (Sigma, UK) supplemented with 10% (vol./vol.) fetal bovine serum (FBS; Gibco, UK) and 1% (vol/vol) pen‐strep. Human islets from ethically approved and next of kin‐consented cadaver pancreas donors were supplied by the King's College Hospital Human Islet Unit, according to previously described protocols 17. Islets (70%–85% purity as assessed by dithizone staining 17) isolated from four donor pancreases were received within 48 hours of pancreas harvest from deceased donors.

### Isolation and Characterization of MSC Populations

**Figure 1:**
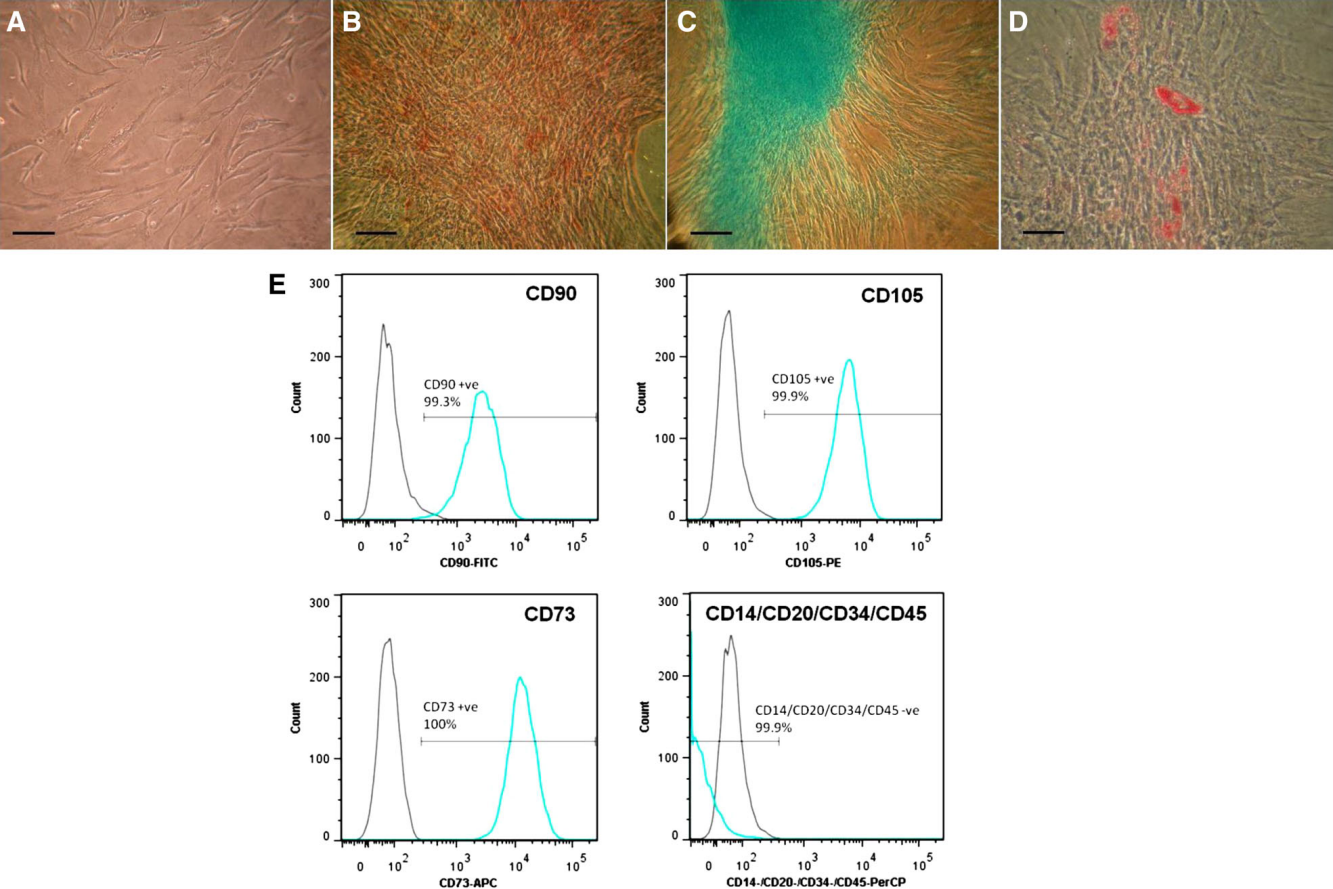
Characterization of human pancreatic mesenchymal stromal cells (MSCs). **(A–D):** Trilineage differentiation potential of human pancreatic MSCs (pMSCs). Prior to differentiation human pMSCs displayed characteristic spindle‐shaped morphology **(A)**. Following 28 days treatment with osteogenic, chondrogenic, or adipogenic differentiation supplements, pMSCs demonstrated trilineage differentiation potential in vitro. Alizarin red staining identified punctate mineral deposition across cell monolayers exposed to osteogenic medium **(B)**. Alcian blue staining detected glycosaminoglycan deposition within cell micromasses directed toward a chondrogenic fate **(C)**. Oil Red O staining detected the formation of lipid droplets in MSCs treated with adipogenic medium **(D)**. Each image is representative of three wells. Scale bar = 100 μm **(A–C)**, 50 μm **(D)**. **(E):** Immunophenotyping human pMSCs. Cultured human pMSCs were analyzed for expression of key cell surface markers by flow cytometry. Negative isotype‐matched controls shown by light gray peaks, marker expression shown by blue peaks. Cells highly expressed markers CD90, CD105, and CD73 (all >99%). Expression of CD14, CD20, CD34, and CD45, markers of monocytes, B cells, and hematopoietic progenitors was absent.

Mouse adipose and kidney MSCs (maMSCs and mkMSCs, respectively) were isolated from male C57Bl/6 mice, characterized, and maintained in culture as adherent monolayers, as previously described 17, 18, 19. Human adipose and bone marrow MSCs (haMSCs and hbmMSCs, respectively) were obtained from commercial sources and maintained according to the suppliers' instructions (Thermo Fisher Scientific R7788110 and Lonza PT‐2501, respectively). Human pancreatic (hp) MSCs were isolated from pancreatic digest and characterized as previously described for mouse MSCs 22. Briefly, digest samples were pelleted by centrifugation (1000*g*, 3 minutes), washed in phosphate‐buffered saline (PBS), resuspended in Dulbecco's modified Eagle's medium (DMEM; Sigma) supplemented with 1% vol/vol pen‐strep, 10% vol/vol MSC‐qualified FBS (Gibco 10500064), seeded into 75 cm^2^ Nunclon tissue culture flasks, and incubated at 37°C, 5% CO_2_. Medium was changed after 24 hours to remove nonadherent cells, and subsequently every 3 days to enrich for an adherent cell population. Cells were subcultured for a further three passages to enrich for adherent spindle‐shaped cells. These populations were characterized by in vitro differentiation into osteoblasts, adipocytes, and chondroblasts and by immunotyping by flow cytometry, as described previously 18, 19. Figure 1A–1D shows trilineage differentiation of hpMSCs maintained in osteogenic, chondrogenic, or adipogenic supplements, and Figure 1E shows immunophenotyping of this hpMSC population. The characterization of MSC populations between passages 3 and 8 in our lab showed similar functional phenotypes, so all cells used in this study were confined to this range of passages.

### Coculture of Islets and MSCs

MSCs (2 × 0^5^ cells) were seeded into 35 mm Nunclon Petri dishes, or six‐well plates, and cultured as described above for 24 hours to form a confluent monolayer. For direct contact islet‐MSC coculture, 100 mouse or human islets were seeded directly onto the MSC monolayers, and the culture medium switched to RPMI‐1640 (Sigma) supplemented with 10% (vol/vol) FBS and 1% (vol/vol) pen‐strep. For indirect contact coculture conditions, inserts (1.0 μm pore, PET membrane, Falcon) were placed into wells of a six‐well plate preseeded with maMSCs, and 100 mouse islets were cultured in the insert. The cocultures were incubated for 72 hours at 37°C, 5% CO_2_. In parallel, control groups of 100 islets alone were incubated in noncoated 35 mm petri dishes or culture inserts in RPMI‐1640 medium. For all experiments, islets were retrieved from the MSC monolayers or Petri dishes by hand picking under a dissecting microscope.

### Assessment of Islet Function In Vitro

Islets were harvested and assessed for glucose‐stimulated insulin secretion by static incubation in buffers supplemented with 2 mM or 20 mM glucose 18, 19. Briefly, islets were preincubated for 2 hours in RPMI containing 2 mM glucose to establish a basal rate of insulin secretion. Groups of three islets were transferred into 1.5‐ml microcentrifuge tubes and incubated at 37°C in a bicarbonate‐buffered physiological salt solution, supplemented with 2 mM CaCl_2_, 0.5 mg/ml BSA, and either 2 or 20 mM glucose. Samples of the incubation medium were taken after 1 hour and stored at −20°C until assayed for insulin content using an in‐house radioimmunoassay 23. Each experimental treatment was tested on 10 separate groups of islets, and experiments were repeated a minimum of 3 times using different islet preparations. For measurements of islet insulin content, groups of 10 islets were incubated for 24 hours in acidified ethanol (52:17:1 absolute ethanol:H_2_O:conc. HCL) at 4°C. In some experiments, a temperature‐controlled (37°C) multichannel perifusion system was used to determine the dynamic pattern of insulin secretion 17. Briefly, groups of 50 islets were placed on 1 μm nylon filters in filter holders (Swinnex, Millipore, Cork, Ireland) and perifused at a flow rate of 0.5 ml/min with the physiological salt solution supplemented with insulin secretagogues. Perifusate samples were collected every 2 minutes and stored at −20°C. Each experimental treatment was tested on four separate groups of islets, and experiments were repeated a minimum of three time using different islet preparations.

### Activation of MSCs

Activation of MSCs was achieved as described previously 4. Briefly, 2 × 10^5^ maMSCs were seeded into Nunclon 35 mm Petri dishes in DMEM (plus supplements, as above) and allowed to adhere for 24 hours. The medium was replaced by DMEM supplemented with interferon‐*γ* (INF−γ) and tumor necrosis factor‐α (TNF‐α; Peprotech, London, United Kingdom; 20 ng/ml each), and the maMSCs were incubated for further 8 hours. MSCs were washed with PBS, trypsinized, and pelleted by centrifugation (1000*g*, 3 minutes), and mRNA was extracted using a commercially available kit (RNeasy mini kit, QIAGEN, United Kingdom) The expression of mRNAs for chemokine (C‐X‐C motif) ligand 9 (CXCL9) and nitric oxide synthase (NOS2) was quantified against glyceraldehyde‐3‐phosphate dehydrogenase mRNA by quantitative real‐time polymerase chain reaction using QIAGEN QuantiTect primers (QT0010124; QT00100275) as described previously 20.

### Statistical Analysis

Differences between treatments were assessed initially using two‐way analysis of variance with post hoc testing using Bonferroni or unpaired Student's *t* test, as appropriate, and considered significant when *p* < .05.

## Results

### Effects of MSC Coculture on Islet Secretory Function

**Figure 2:**
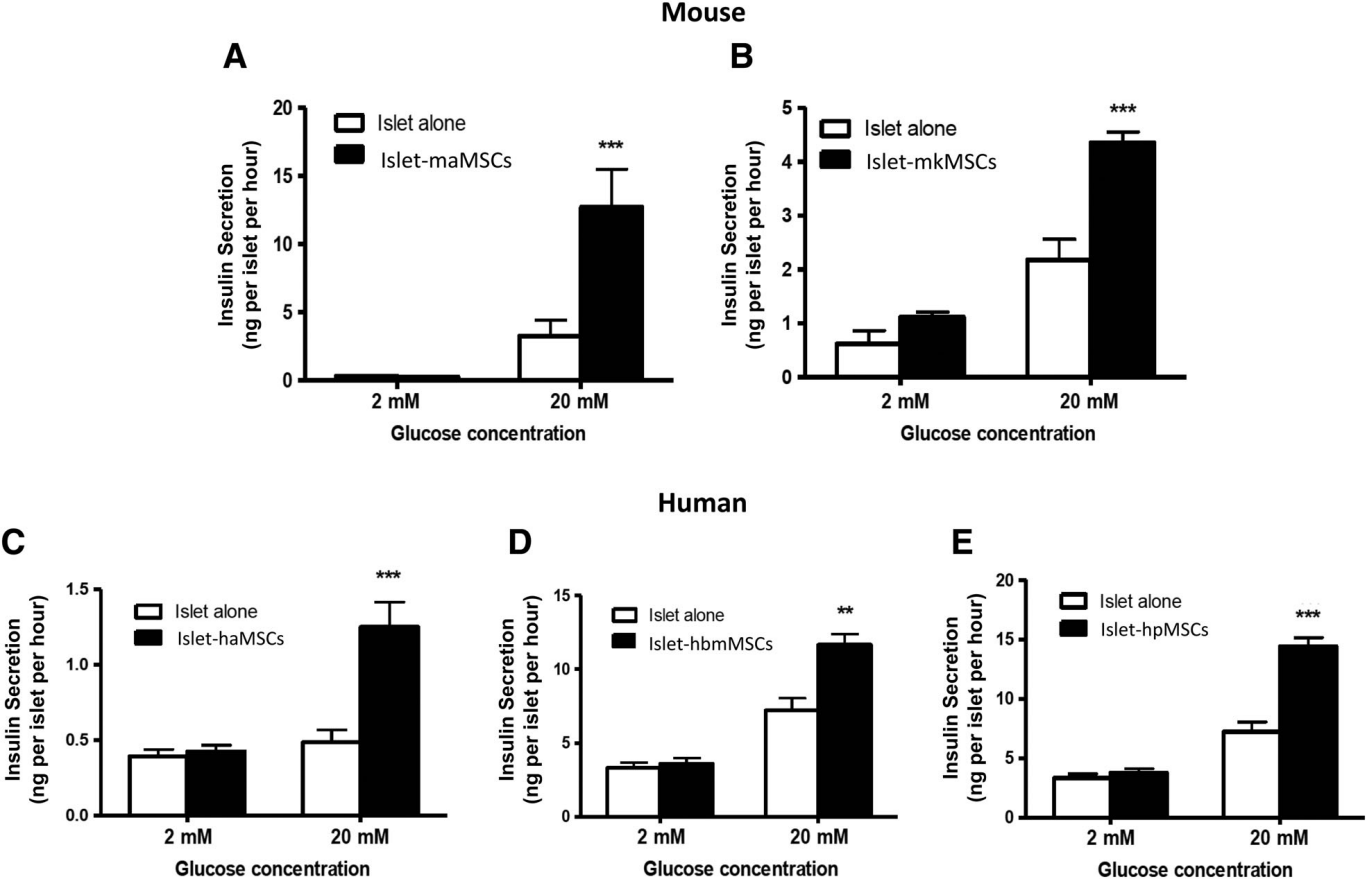
Mesenchymal stromal cells enhance glucose‐induced insulin secretion from mouse and human islets. Coculture (72 hours) with **(A)** maMSCs (solid bars) or **(B)** mkMSCs (solid bars) significantly increased insulin secretion from mouse islets in response to a subsequent exposure to 20 mM glucose when compared with untreated mouse islets (open bars). Similarly, coculture of human islets with **(C)** haMSCs, **(D)** hbmMSCs, or **(E)** hpMSCs (solid bars) significantly enhanced glucose‐induced insulin secretion compared with untreated human islets (open bars). Graphs show one experiment typical of three or four separate experiments using different islet preparations. Bars show means + SEM; *n* = 10; **, *p* < .01; ***, *p* < .001 versus untreated islets. Abbreviations: haMSCs, human adipose mesenchymal stromal cells; hbmMSCs, human bone marrow mesenchymal stromal cells; hpMSCs, human pancreatic mesenchymal stromal cells; maMSCs, mouse adipose mesenchymal stromal cells; mkMSCs, mouse kidney mesenchymal stromal cells.

Initial experiments were performed to assess whether MSCs isolated from different tissues had similar effects on islet function and to determine whether mouse and human islets responded in similar ways to coculture with MSCs. These experiments used a direct contact coculture configuration and an incubation time of 72 hours, parameters which we had previously reported to enhance β‐cell function by MSCs 17. Figure 2A, 2B shows that coculture of mouse islets with maMSCs or mkMSCs caused a significant increase in glucose (20 mM)‐induced insulin secretion when compared with untreated control islets. In each of three separate experiments using different mouse islet preparations, those which had been pretreated with maMSCs secreted significantly (*p* < .01) more insulin in response to 20 mM glucose than those pretreated with mkMSCs, reflected by the different scaling of the *y* axes of the panels in Figure 2A, 2B. The increased insulin release from maMSC‐pretreated islets was not secondary to alterations in islet insulin content (control untreated, 43 ± 3 ng insulin per islet; maMSC pretreated 36 ± 6, *n* = 3, *p* > .2), as has been reported previously 17, 19. Human MSCs exerted similar beneficial effects on glucose‐induced insulin secretion from human islets, as shown in Figure 2C–2E. Isolated human islets are inherently more variable than mouse islets in their insulin secretory responses to glucose, but the effects of MSCs to enhance insulin secretion in the presence of 20 mM glucose were observed in islets isolated from four separate donors. Thus, MSCs isolated from four different tissues had qualitatively similar effects on glucose‐induced insulin secretion when maintained in a direct contact configuration; MSCs exerted beneficial effects on both mouse and human islets; and adipose‐derived MSCs were consistently more effective, at least with mouse islets. Subsequent experiments to further characterize islet–MSC interactions were therefore focused on mouse islets and maMSCs.

### Effect of Protocol Configuration on MSC–Islet Interactions

We compared two commonly used protocol configurations for studying MSC–islet interactions: direct contact, in which the islets are cultured in suspension on top of an adherent MSC monolayer, and indirect contact, in which the islets were separated from the MSC monolayer by a porous membrane 24. Figure 2A shows that direct contact between mouse islets and maMSCs enhanced subsequent glucose‐induced (20 mM) insulin secretion. In contrast, indirect coculture experiments ran in parallel showed no enhancement of glucose‐induced insulin secretion. Thus, in three separate experiments using different mouse islet populations, indirect coculture had no significant effect (*p* > .2) on basal (2 mM glucose) insulin secretion; no significant effect (*p* > .2) on glucose‐induced insulin secretion in two experiments, and a significant inhibition (*p* < .01) of glucose‐induced insulin in one experiment (20 mM glucose: MSC precultured islets 59% ± 5% controls, *n* = 10, *p* < .01). Therefore, subsequent experiments were performed using the direct contact coculture configuration.

### Effect of MSC Coculture Length on Islet Secretory Function

**Figure 3:**
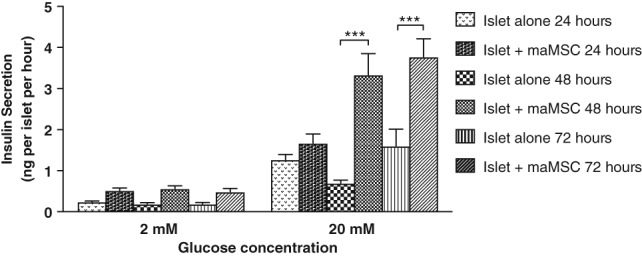
Time course of effects of mesenchymal stromal cell (MSC) coculture on glucose‐induced insulin secretion. Insulin secretion by mouse islets was measured at substimulatory (2 mM) and stimulatory (20 mM) glucose concentrations after coculture with maMSCs for 24, 48, or 72 hours, as shown by the key. Data are presented as mean + SEM, *n* = 10 observations in one experiment typical of three separate experiments using different islet preparations. ***, *p* < .001, versus non‐MSC‐treated islets controls at appropriate time points. Abbreviation: maMSCs, mouse adipose mesenchymal stromal cells.

The effects of MSC coculture on mouse islet secretory function were assessed by measuring basal (2 mM glucose)‐ or glucose (20 mM)‐induced insulin release after periods of 24, 48, or 72 hours. Figure 3 shows that coculture for 24 hours had no significant effect on glucose‐induced insulin secretion, whereas significant increases were observed in islets maintained in coculture with maMSCs for 48 or 72 hours, with no significant differences between these time points. MSC coculture did not significantly increase basal (2 mM glucose) insulin secretion (*p* > .2). These effects were observed in three separate experiments using different mouse islet populations. Thus, the effects of MSCs on β‐cell secretory function are relatively slow to develop, requiring a minimum coculture period of 48 hours.

### Effects of MSC Activation on MSC–Islet Interactions

**Figure 4:**
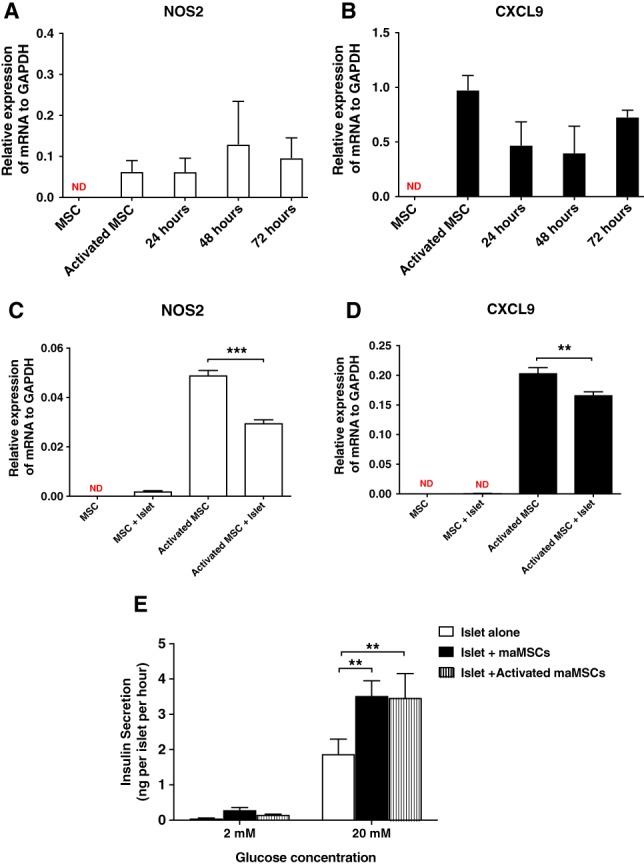
Activation of maMSCs. **(A, B):** Cytokine‐induced activation of maMSCs. maMSCs were cultured in the absence or presence of interferon‐γ (20 ng/ml) and tumor necrosis factor‐α (20 ng/ml) for 8 hours and NOS2 **(A)** and CXCL9 **(B)** mRNAs were measured 24, 48, and 72 hours after the removal of cytokines. Data are expressed as mean + SEM, *n* = 3 in one experiment representative of three separate experiments. **, *p* < .001; ND, not detectable. **(C, D):** Effects of islet coculture on maMSC activation. Quiescent or cytokine‐activated maMSCs were cocultured with mouse islets for 72 hours, followed by the measurement of NOS2 **(C)** and CXCL9 **(D)** mRNAs in the maMSC populations. Data are expressed as mean + SD; *n* = 3 observations in one experiment representative of three separate experiments. **, *p* < .01. **(E):** Effects of maMSC activation on glucose‐stimulated insulin secretion from mouse islets. Insulin secretion at substimulatory (2 mM) and stimulatory glucose concentration (20 mM) from islets alone (white bar), islets cocultured with maMSCs (black bar), and islets cocultured with activated maMSCs (hatched bar). Data are presented as mean + SEM; *n* = 10 observations. ***, *p* < .001; **, *p* < .01; *, *p* < .05. Abbreviations: GAPDH, glyceraldehyde‐3‐phosphate dehydrogenase; maMSCs, mouse adipose mesenchymal stromal cells; MSCs, mesenchymal stromal cells.

Activation of MSCs by an inflammatory environment is associated with their adopting an anti‐inflammatory phenotype (MSC2 cells), as evidenced by the expression of CXCL9 and NOS2 4. Under unstimulated conditions, the expression of mRNAs for CXCL9 and NOS2 was undetectable in maMSCs. In contrast, maMSCs incubated in vitro (8 hours) with the inflammatory cytokines INF‐γ and TNF‐α expressed high levels of NOS2 and CXCL9 mRNAs (0.113 ± 0.01 vs. 0.0001 ± 5.543e‐005, 0.8 ± 0.2 vs 0.0002 ± 0.0002, respectively), which is consistent with an activated profile. The cytokine‐induced activation of maMSCs was maintained for at least 72 hours after removal of the cytokines, as demonstrated by the maintained levels of CXCL9 and NOS2 mRNA expression (Fig. 4A, 4B). The coculture of islets with unstimulated maMSCs (72 hours) did not influence their activation status, as assessed by NOS2 and CXCL9 mRNA levels. However, coculturing islets with cytokine‐activated MSCs did cause a small, albeit significant, reduction in the expression of both mRNAs (Fig. 4C, 4D). Cytokine‐induced activation of maMSCs had no effect on their ability to enhance glucose‐induced insulin secretion, as shown in Figure 4E. Direct coculture of mouse islets with maMSCs significantly enhanced their subsequent insulin secretory responses to 20 mM glucose, as previously observed (Figs. 2, 3), but there were no differences in basal (2 mM glucose) nor stimulated (20 mM glucose) insulin secretion from islets cocultured with unstimulated or activated maMSCs (Fig. 4E). Thus, the beneficial effects of MSCs on β‐cell insulin secretory function are independent of their activation status.

### Effects of MSCs on Insulin Secretion in Response to Non‐Nutrients

**Figure 5:**
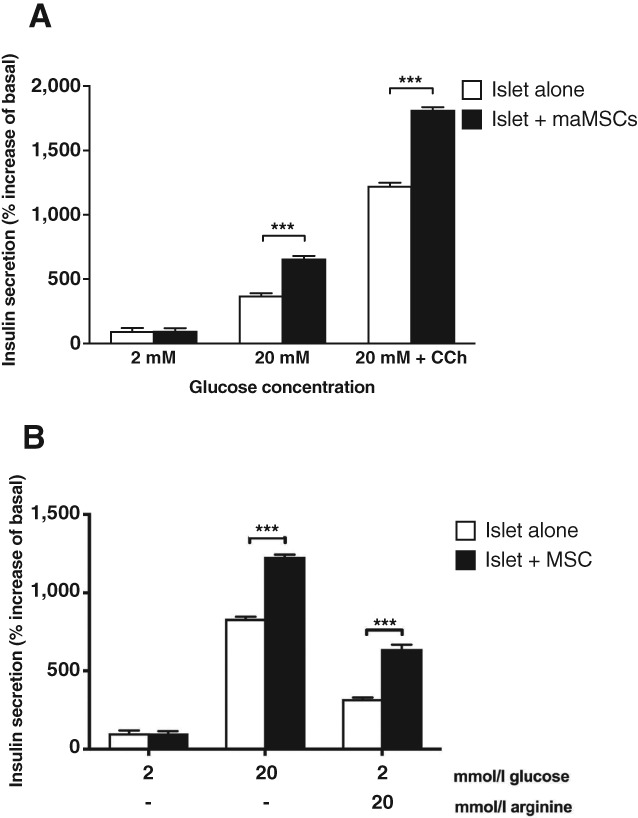
Effect of mesenchymal stromal cells coculture on insulin secretion in response to non‐nutrients mouse islets were cultured for 48 hours either alone (white bars) or with maMSCs (black bars), followed by measurement of insulin secretion in response to **(A)** 2 mM glucose, 20 mM glucose, and 20 mM glucose + 500 μM carbachol; or **(B)** 2 mM glucose, 20 mM glucose, and 20 mM arginine. Data are presented as increase in insulin secretion over basal (2 mM glucose), mean + SEM; *n* = 10. ***, *p* < .001. Abbreviation: maMSCs, mouse adipose mesenchymal stromal cells.

Islet β‐cells use different mechanisms to recognize and respond to nutrient and non‐nutrient stimuli 25, 26. Nevertheless, most studies to date have focused solely on MSCs influencing β‐cell responses to glucose, the main nutrient secretagogue in mammals. Here, we have investigated two non‐nutrient secretagogues, the nicotinic cholinergic receptor agonist carbachol, and the amino acid arginine. Figure 5A shows that islets cocultured with maMSCs show significantly enhanced insulin secretory responses both to 20 mM glucose alone and to 20 mM glucose in the presence of carbachol (500 μM). Figure 5B shows that arginine (20 mM) was sufficient to stimulate insulin secretion at basal glucose (2 mM) and that preculture with maMSCs significantly increased insulin secretory responses to arginine alone and to glucose alone (20 mM), as previously observed (Figs. 2, 3). Thus, coculture with MSCs enhances β‐cell insulin secretory responses to both nutrient and non‐nutrient secretagogues.

### Effects of MSCs on Biphasic Insulin Secretion

**Figure 6:**
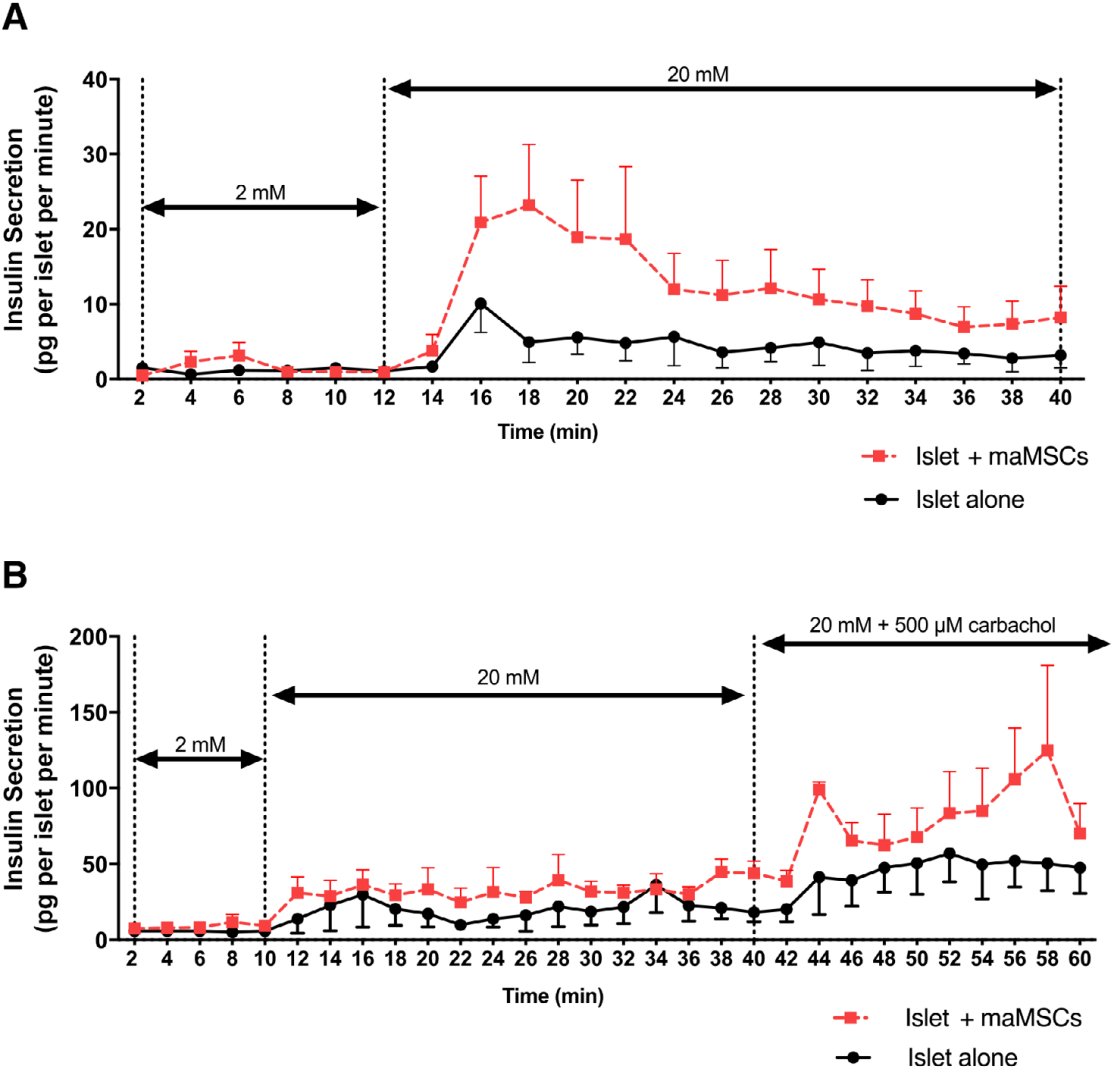
Effect of mesenchymal stromal cells on dynamic insulin secretion from mouse islets. Mouse islets cultured for 48 hours either alone (black, solid line) or with maMSCs (red, dotted line) were perifused with solutions containing (A) 2 or 20 mM glucose; or (B) 20 mM glucose + 500 μM carbachol. Points show mean ± SEM; *n* = 4 observations in one experiment typical of three separate experiments using different islet preparations. Abbreviation: maMSCs, mouse adipose mesenchymal stromal cells.

Glucose‐induced insulin secretion shows a distinctive temporal pattern, with a rapidly rising initial peak (first phase), followed by a maintained plateau (second phase), with both phases being regulated by different β‐cell intracellular mechanisms 27. Dynamic measurements of insulin secretion from mouse islets demonstrated that preculture with maMSCs enhanced both phases of the secretory response to glucose (20 mM), as shown in Figure 6A. Perifusion of the islets with a substimulatory concentration of glucose (2 mM) established a low, stable rate of insulin secretion. Switching to a maximum stimulatory concentration of glucose (20 mM) initiated a rapid peak (2–4 minutes) in insulin secretion (first phase), followed by a maintained elevation in insulin secretion in response to 20 mM glucose (second phase). Islets which had been cocultured with maMSCs secreted significantly more insulin throughout the exposure to 20 mM glucose, indicating enhancement of both first and second phases of the secretory responses. In a similar experiment (Fig. 6B), the perifused islets were subsequently exposed to carbachol in the presence of 20 mM glucose, which further and substantially enhanced the insulin secretory response to 20 mM glucose alone (note different y axes in panels A and B). Islets which had been cocultured with maMSCs secreted significantly more insulin throughout the exposure to 20 mM glucose and to 20 mM glucose plus carbachol, confirming the enhancement of secretory responses to both nutrient and non‐nutrients (see Fig. 5).

## Discussion

There is accumulating evidence that MSCs can exert beneficial effects on islet β‐cell function and survival, thus improving the outcomes of islet transplantation in experimental models of T1D. The well‐documented anti‐inflammatory and immunosuppressant effects of MSCs are likely to improve graft survival in vivo by modifying the host environment and in vitro studies have also demonstrated direct effects of MSCs on islet β‐cells to improve their insulin secretory capacity 17, 19, 28 and to protect them from apoptotic stimuli 20. This raises the possibility of enhancing islet graft functional survival by treating the islets with MSCs before transplantation 24, so avoiding the logistical, safety, and regulatory concerns of codelivering MSCs in clinical islet transplantation protocols. To date, experimental studies have used a range of different MSCs and in vitro culture conditions 8, 12, 20, 29, 30, but the effective translation of these experimental observations into improved clinical protocols will require consensus about how best to use MSCs to support graft function. In the current study, we have investigated a number of parameters to optimize the beneficial effects of in vitro coculture of islets with MSCs.

Our observations that MSCs derived from four different tissues had similar qualitative effects to enhance insulin glucose‐induced secretion without affecting basal secretion suggest that this is a general property of MSCs. Our measurements showed that the MSC‐dependent enhancement of insulin secretion was not secondary to increased insulin content, confirming previous observations in mouse 17, 19 and human 17 islets, consistent with an upregulation of the β‐cell secretory response to elevated glucose, the most physiologically important initiator of insulin secretion in mammals 27. Importantly, our experiments using human islets confirm and extend previous studies 17, 19 to demonstrate that human MSCs from a variety of tissues have the ability to enhance human β‐cell function. Human islets are much more variable than mouse islets, and factors such as donor age, body mass index, and health status, as well as duration of donor pancreas cold ischemia time, are known to affect human islet function 31. Nevertheless, the available evidence confirms the potential benefits of incorporating MSCs into clinical islet transplantation protocols. The optimal source of MSCs for clinical use in islet transplantation will likely be a balance between functional phenotype and accessibility. Bone marrow‐ and adipose‐derived MSCs are commonly used experimentally because of the accessibility of the tissues for autologous grafts. Our early in vivo studies used kidney‐derived MSCs because mouse islets are commonly transplanted below the kidney capsule 9, 18, 22, and pancreatic MSCs can be derived from pancreatic digests used to isolate donor islets. We suggest that autologous MSCs isolated and expanded from host adipose biopsies may offer the simplest source in a clinical setting.

Several different configurations have been used to facilitate islet–MSC interactions during coculture in vitro, including direct contact of islets on an adherent monolayer of MSCs 17, 28; the formation of composites by suspension culture of islets and MSCs 2, 8, 32; and indirect coculture conditions, where islets and MSCs are physically separated by porous barriers to prevent contact but allow the diffusion of soluble mediators 28. In agreement with our study, direct contact coculture of islets with MSCs has consistently been reported to improve glucose‐stimulated insulin secretion, whereas noncontact coculture systems are more variable, with some reports of enhanced insulin secretion 8, 33 and others of no effect 18, 28, 34. There is convincing evidence that at least some of the beneficial effects of MSCs on β‐cell function are mediated via secreted molecules 17, 20, 35, which should be able to access the islet cells in the indirect coculture systems. These in vitro effects of MSC‐derived molecules on islet function persist for several days after treatment 35, and translate into improved islet graft in vivo in a mouse model of diabetes, as assessed by glycemic control 20, 35. However, the diffusion distances and consequent dilution of MSC‐derived soluble mediators in such configuration may prevent effective concentrations reaching the islets, which may explain some of the reported inconsistencies between studies 8, 18, 28, 33, 34. The effectiveness of direct contact coculture may also reflect the importance of interactions between islet cells and MSC‐derived ECM 17, which can influence β‐cell function both via direct contact and by acting as a local reservoir for biologically active MSC‐derived secretory products 24, thus maintaining their high local concentrations in the vicinity of the islet cells. We suggest that direct contact coculture of islets with MSCs prior to transplantation is likely to be the most effective configuration for improving human islet transplantation protocols.

Most studies which report the coculture of islets with MSCs 18, 19, 22, 28 or with MSC‐derived secretory products 17, 20 use incubation periods greater than 48 hours, although the rationale for this time frame is rarely justified. Our results demonstrate that the effects of MSC on β‐cell function are relatively slow in onset, requiring 48–72 hours to develop fully. This might seem unexpected, particularly for the effects mediated by MSC‐derived soluble molecules acting on β‐cell surface receptors 17, 20, but it may reflect the time required for MSCs to generate sufficient ECM or effective local concentrations of soluble mediators to influence β‐cell function. Alternatively, the onset of functional changes in the β‐cells may require modifications in gene expression which can take time to manifest as phenotypic changes. For example, it is well established that interactions with ECM induce numerous modifications in the expression of genes influencing β‐cell survival, growth, and differentiation 24, 36 and that MSC‐derived secretory factors modify gene expression in their target cells 37. MSC‐mediated changes in β‐cell gene expression is also consistent with the sustained effects of MSC‐derived secretory factors on β‐cell function in vitro, some of which were maintained for up to 72 hours after the removal of the factors from the culture medium 35. Irrespective of the underlying mechanisms, the incorporation of MSC pretreatment into human islet transplantation protocols will require a minimum of 48 hours coculture period before implantation. The practice of centralized human islet isolation facilities with distribution to remote transplantation centers, along with the requirement for pretransplantation assessment of islet function and sterility, do allow for such a coculture period.

Activation or “licensing” of MSCs is thought to be important for their adopting an anti‐inflammatory phenotype 4, 21, 38, 39, 40. Our study confirms that in vitro exposure of MSCs to inflammatory cytokines induces their prolonged activation but coculture with islets was not alone sufficient to induce MSC activation. These observations imply that activation of MSCs is likely to be a property of the inflammatory host niche rather than of the islet graft and most likely mediated by the innate immune response to the graft. Delivery of islet grafts via the clinically preferred intraportal route will expose them to both the instant blood‐mediated inflammatory response and to the endogenous liver immune system 41, whose inflammatory response to the graft may be exacerbated by the presence of acinar tissue in the less pure human islet preparations 42. Further studies are required to determine whether MSCs, activated or unactivated, exert protective effects on islets lodged in the hepatic microvasculature. We also demonstrated that MSC‐mediated effects on β‐cell secretory function were independent of MSC activation. This is consistent with MSCs influencing graft functional survival through two independent mechanisms: direct effects on β‐cell function, which do not require MSC activation and improvement of the host niche by reducing inflammation, which may require MSC activation.

Previous studies of MSCs and β‐cell secretory function have focused on their enhanced secretory responses to glucose 18, 19, 43 17, 28. We demonstrate here that MSC pretreatment also enhances insulin secretion in response to non‐nutrient and receptor‐mediated agonists. The mechanisms of glucose recognition by β‐cells are well understood: glucose and other nutrients initiate insulin secretion by increasing metabolic fluxes and ATP generation, leading to β‐cell depolarization and Ca^2+^ influx through voltage‐dependent Ca2+ channels, which triggers the exocytotic release of insulin 27. In contrast, carbachol and other receptor‐mediated agonists do not initiate insulin secretion but potentiate responses to nutrients via well‐characterized intracellular pathways 27, whereas the cationic amino acid arginine is thought to stimulate insulin secretion primarily by inducing depolarization upon its transport into the β cell 44. Our observation that MSC coculture enhanced β‐cell responses to all these stimuli suggests an overall upregulation of the exocytotic process rather than specific effects on signal recognition and intracellular transduction cascades. This is in accordance with the recent report that coculture of human hepatocytes with MSCs enhanced their secretion of albumin into the medium 45, a process which occurs by constitutive exocytosis and is therefore independent of signal recognition and stimulus‐response coupling cascades. Thus, MSC pretreatment may facilitate graft function in vivo when β‐cells are required to respond not only to glucose but also to potentially hundreds of receptor‐operated signals 46.

## Conclusion

In summary, the results of this and other studies suggest that coculture of islets with MSCs pretransplantation has the potential to improve overall graft insulin secretory function. Direct contact coculture with autologous, adipose‐derived MSCs for a minimum of 48 hours before implantation is likely to be an effective addition to human islet transplantation protocols.

## Author Contributions

A.A.A.: conception/design, collection and/or assembly of data, data analysis and interpretation, manuscript writing; A.V.‐S., C.L.R.: collection and/or assembly of data, data analysis and interpretation, manuscript editing; P.K.D.: collection and/or assembly of data, data analysis and interpretation; G.‐C.H., P.C.: collection and/or assembly of data; A.J.F.K.: conception/design, data analysis and interpretation, manuscript editing; P.M.J.: conception/design, data analysis and interpretation, manuscript writing, final approval of manuscript.

## Disclosure of Potential Conflicts of Interest

The authors indicated no potential conflicts of interest.
